# Prodromal Dementia With Lewy Bodies and Recurrent Panic Attacks as the First Symptom: A Case Report

**DOI:** 10.3389/fneur.2022.839539

**Published:** 2022-04-13

**Authors:** Alberto Jaramillo-Jimenez, Yinbing Ying, Ping Ren, Zhan Xiao, Qian Zhang, Jian Wang, Han Rong, Miguel Germán Borda, Laura Bonanni, Dag Aarsland, Donghui Wu

**Affiliations:** ^1^Centre for Age-Related Medicine (SESAM), Stavanger University Hospital, Stavanger, Norway; ^2^Faculty of Health Sciences, University of Stavanger, Stavanger, Norway; ^3^Grupo de Neurociencias de Antioquia, School of Medicine Medellín, Universidad de Antioquia, Medellín, Colombia; ^4^Grupo Neuropsicología y Conducta, School of Medicine Medellín, Universidad de Antioquia, Medellín, Colombia; ^5^Shenzhen Kangning Hospital, Shenzhen Mental Health Center, Shenzhen, China; ^6^Semillero de Neurociencias y Envejecimiento, Ageing Institute, Medical School, Pontificia Universidad Javeriana, Bogotá, Colombia; ^7^Department of Medicine and Aging Sciences, University G. D'Annunzio of Chieti-Pescara, Chieti, Italy; ^8^Department of Old Age Psychiatry, Institute of Psychiatry, Psychology, and Neuroscience, King's College London, London, United Kingdom

**Keywords:** dementia with Lewy bodies, panic attacks, case report, prodromal dementia with Lewy bodies, neuropsychiatric symptoms

## Abstract

Psychiatric-onset dementia with Lewy bodies (DLB) might include symptoms of depression, hallucinations, anxiety, and apathy. Here, we report a patient with DLB with recurrent panic attacks as her first symptom 5 years before a biological-based diagnosis of probable DLB. We provide an extended description of the clinical presentation and course from psychiatric-onset DLB to dementia in an 83-year-old woman. This case illustrates the common misdiagnosis of DLB and the delay of having a detailed clinical and biomarker assessment for structured diagnosis. With a detailed description of the clinical presentation of this case, the empirical treatment strategies, and the patient perspectives, we aim to make clinicians aware of panic attacks within the psychiatric-onset DLB.

## Introduction

Dementia with Lewy Bodies (DLB) is a frequent cause of neurodegenerative dementia, but is often under or misdiagnosed (1). Recently, the focus on the prodromal stage of DLB is increasing, and the first consensus research criteria have been proposed (2). The suggested prodromal phenotypes include mild cognitive impairment (MCI), delirium-onset, and psychiatric-onset. In the latter, depression, hallucinations, anxiety, and apathy are among symptoms suggested as prodromal DLB, but scarce evidence impedes formal criteria. Thus, further investigations are recommended (2).

One preliminary case series found an increased frequency of anxiety symptoms before diagnosis in patients with DLB compared to prodromal Alzheimer's Disease. However, the clinical diagnosis of anxiety was not structured nor quantified (3). Particularly, prodromal panic attacks have not been explored in detail in DLB. In addition, therapeutic interventions, clinical presentation, and progression have not been reported.

Here, we report a patient with DLB with recurrent panic attacks as her first symptom, providing an extended description of the clinical presentation of psychiatric-onset DLB with an operationalized and biological-based diagnosis approach.

## Patient Information and Chief Complaint

An 83-year-old a highly educated woman was admitted to the geriatric-psychiatry department at Shenzhen Kangning Hospital. She presented with cognitive decline and functional impairment in activities of daily living before the hospitalization, complex visual hallucinations (i.e., seeing children and other people at home), bradykinesia, and hypophonia.

### Medical History

Neurodevelopment was normal. She graduated from college and worked as a teacher before retirement at 60-years-old. There was no history of psychiatric symptoms or family history of psychiatric disorders or dementia.

**Figure 1:**
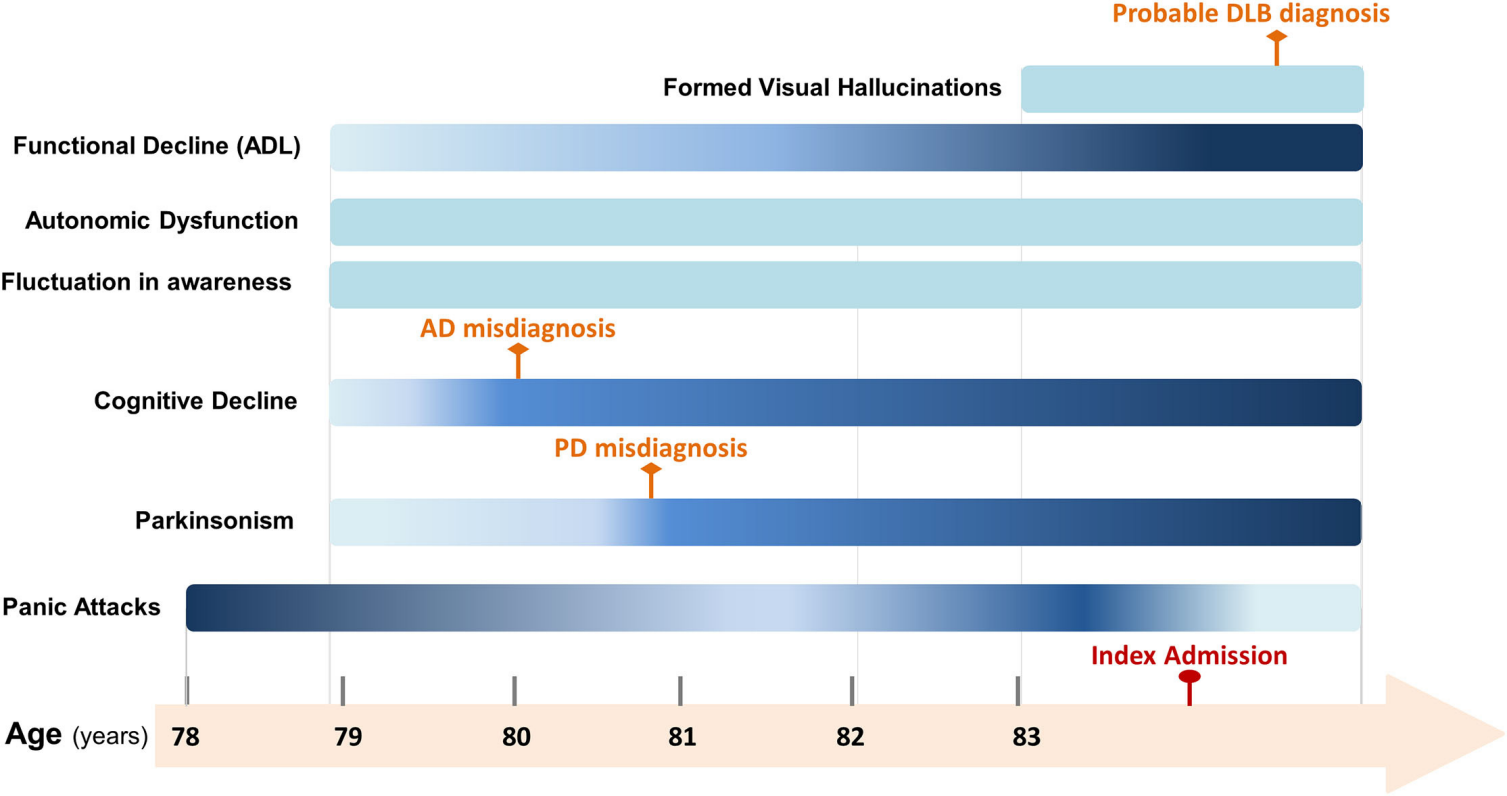
Timeline of relevant symptoms presented from psychiatric-onset prodromal dementia with Lewy bodies (DLB) to DLB in moderate dementia stage. Gradient-colored symptoms vary in severity. Darker colors indicate more severity while light colors indicate less severity. Solid-colored symptoms did not vary in severity. AD, Alzheimer's Disease; PD, Parkinson's Disease; DLB, Dementia with Lewy Bodies; ADL, Activities of daily living.

A graphical timeline with the most relevant symptoms is shown in Figure 1.

Her present illness started with panic attacks at age 78, consisting of abrupt surges of intense emotional discomfort accompanied by abdominal distress (i.e., heartburn), shortness of breathing, chest discomfort (i.e., chest tightness), feelings of choking, and fear of dying or being seriously ill. The episodes, lasting around 1 h, were recurrent (3–4 per day) and were triggered by situations, such as waiting in line or seeing traffic. Flupentixol-Melitracen (21 mg/day) was prescribed but caused nervousness and hand trembling. The frequency of symptoms decreased with Sertraline (50 mg/day) and Mirtazapine (15 mg/day). At the end of that year, she developed parkinsonism (bradykinesia, hands tremble, hypophonia, and hypomimia), reported fluctuations in arousal (described as lethargic episodes), two episodes of syncope, marked constipation, and subjective decline in language and memory.

At the age of 80, cognitive decline and parkinsonism became prominent. Structural magnetic resonance image (MRI) showed diffuse brain atrophy with no vascular injury, leading to a misdiagnosis of possible Alzheimer's Disease treated with Donepezil. She ceased this medication because of heartburn and physical discomfort, to which rivastigmine transdermal patches were prescribed. After a few months, she was re-admitted due to increased parkinsonism and was again misdiagnosed, but with Parkinson's Disease. Levodopa + Benserazide was prescribed, causing significant improvement of tremor and hypomimia for less than one year.

During the 2 years before index admission, bradykinesia, hypophonia, and cognitive decline (in language and memory) gradually worsened. Panic attacks continued at a lower frequency (1 episode per day, 1 h each), and postprandial hypotension episodes (systolic blood pressure drop of 40 mmHg after eating) were also evidenced.

At 83 years old, treatment with GV-971 (Sodium Oligomannate) was prescribed for 2 months with no subjective effect and, thus, was discontinued. Two months prior to the index admission, function worsened (i.e., problems for food preparation and housekeeping) and the patient experienced an increase in formed visual hallucinations (i.e., people around her in different home settings). Increased daytime sleepiness was also reported. She received Memantine, which she decided to discontinue due to increased anxiety symptoms.

Causes of secondary dementia and delirium were ruled out.

### Clinical Findings at the Index Admission

On physical examination, she had hypomimia. She was wheelchaired and needed help to stand up, of which she was unsteady. She exhibited a slow shuffling gait, poor balance, flexed posture, and mild stiffness in limbs. During the mental examination, the patient was disoriented in time and place, hypophonic, with slow verbal responses, passive contact, with the poverty of speech (laconic speech), with hallucinatory behavior, apathetic, with slow psychomotor activity, and had lack of insight. Mini-Mental State Examination score was 13/30, 12-item Neuropsychiatric Inventory total score was 51/144 (caregiver disruption score 26/60), and Geriatric Depression Scale score was 16/30.

### Biomarkers

**Figure 2:**
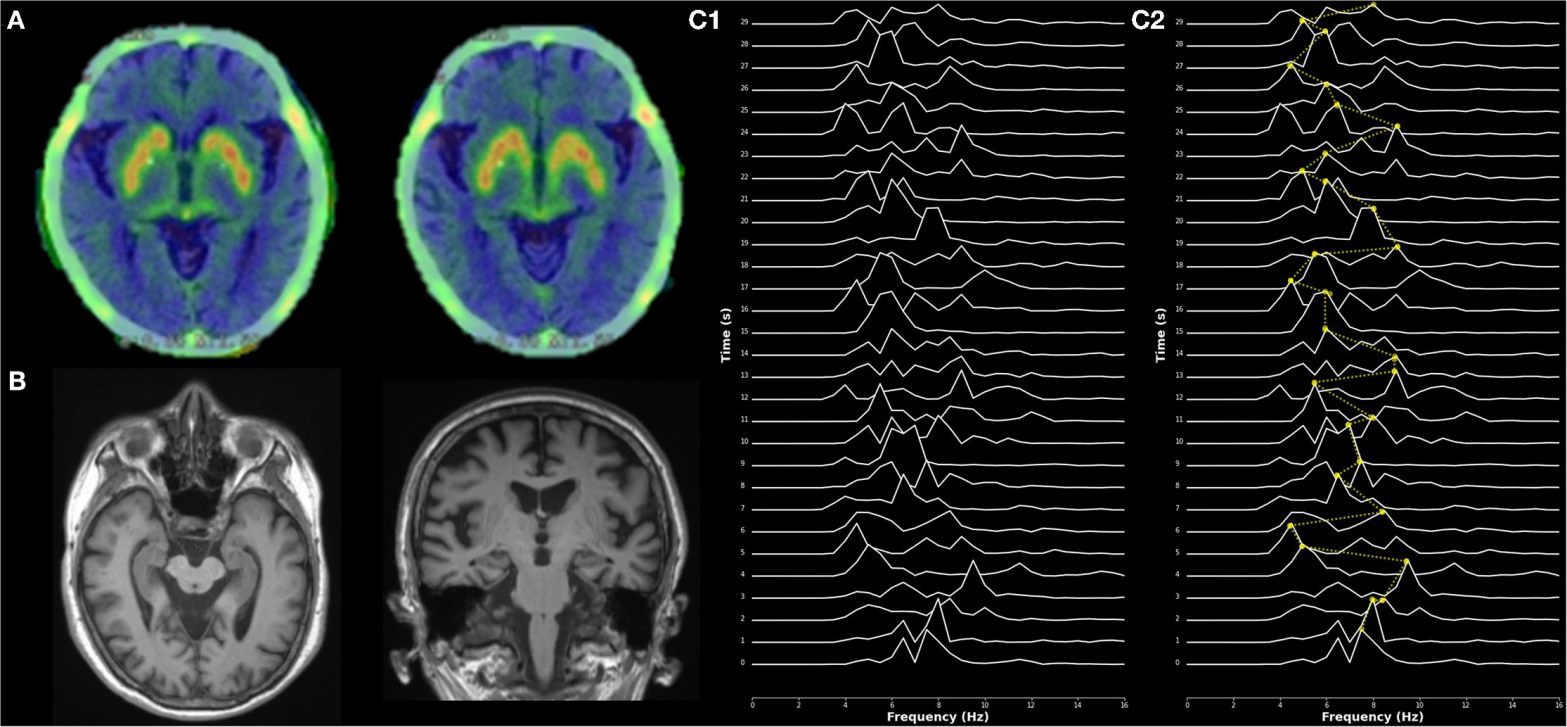
Imaging and electrophysiological findings. **(A)** ^18^F-dihydroxyphenylalanine ^18^F-DOPA PET showed reduced dopamine uptake in the nigrostriatal regions. **(B)** Axial (left) and coronal (right) MRI planes of hippocampal structures in T1 sequence, showing relative sparing of hippocampi. **(C)** Electroencephalogram (EEG)—Compressed spectral arrays (CSA) showing stacked plots of the power spectral density for each 1-s epoch. Frequencies lower than theta were filtered as could be affected by artifacts. **(C1)** CSA from derivation O1 (left occipital) during the eyes-closed condition. **(C2)** Interpretation of CSA from derivation O1 (left occipital). Yellow dots illustrate the dominant frequency (frequency with a maximum power peak) in each 1-s epoch. The dotted yellow line depicts dominant frequency variability across epochs. Dominant frequency in each epoch variates from the alpha band (at 9 Hz) to the pre-alpha/fast-theta band (at 6–7 Hz). Dominant frequency variability of 3 Hz is represented.

An extensive biomarker assessment was conducted (4). The ^18^F-dihydroxyphenylalanine (DOPA) Positron Emitted Tomography (PET) demonstrated reduced uptake in the nigrostriatal pathway, but increased uptake in the pons and medulla oblongata (Figure 2A). Structural MRI revealed mild diffuse cortical atrophy, but relative sparing of hippocampi in the T1 sequence (Figure 2B) as defined by medial temporal lobe atrophy score (5), and minimal white matter vascular lesions in FLAIR and T2 sequences according to Fazekas scale (6). Resting-state electroencephalogram (EEG) in wakefulness with eyes closed was analyzed using compressed spectral arrays (CSA) which represent the power spectral density of EEG signals across each 1-s epoch (7). The CSA of occipital derivations showed a highly variable dominant frequency (between alpha at 9 Hz and pre-alpha/fast-theta at 6–7Hz), with variability of 2–3 Hz. The latter defines a typical pattern of early DLB (8) at the index admission (Figure 2C). One month after the index admission, a 24-h EEG was performed, during which the patient experienced two panic attacks. The EEG result did not show epileptiform activity.

Apolipoprotein-E (APOE) genotype was ε3/ε4. Cerebrospinal fluid (CSF) showed reduced amyloid-beta 1–42 (200.85 pg/ml, reference: 550 pg/ml) and slightly increased phosphorylated tau (61.92 pg/ml, reference: 61 pg/ml), with normal total tau (395.96 pg/ml, reference: 452 pg/ml).

### Diagnosis

The previous diagnosis of Parkinson's Disease was not considered appropriate since it did not fulfill the Movement Disorder Society criteria due to the presence of symptoms such as lack of laterality, presence of autonomic symptoms, and early dementia (considered initially as possible Alzheimer's Disease) (9).

The patient fulfilled both central and two core clinical features for probable DLB (i.e., dementia accompanied by recurrent formed visual hallucinations, and parkinsonism) with possible variation in awareness and various supportive clinical features (i.e., syncope, postural instability, autonomic dysfunction with marked constipation, anxiety, and depression symptoms). No other diagnostic criteria for DLB were present. The clinical diagnosis was supported by several biomarkers (i.e., reduced dopamine synthesis and uptake in the nigrostriatal pathway in ^18^F-DOPA PET, EEG prominent periodic fluctuations in the pre-alpha/fast-theta range, and relative sparing of hippocampus on MRI) (1). The CSF results suggested additional Alzheimer-type pathology, which is found in the majority of patients with DLB (2), as well as those with any APOE ε4 allele (10).

In addition, her prodromal psychiatric symptoms were clustered within the panic attack specifier of the Diagnostic and Statistical Manual of Mental Disorders—Fifth Edition (DSM-5) (11) and were considered as a psychiatric-onset DLB (2). The differential diagnosis of panic attacks with non-convulsive epileptic phenomena (i.e., partial seizures) was also considered. However, several clinical characteristics supported a non-epileptic origin, including attacks duration (up to 1 h), triggering situations that would unlikely trigger partial seizures (waiting in a line), and the partial response to antidepressant drugs at the onset of panic attacks (12). Other potential origins for panic attacks were discarded, such as potential vascular lesions (not observed in MRI) in the absence of cardiometabolic risk factors. Alzheimer-type pathology, evidenced in CSF, could be linked to panic attacks. Evidence has shown positive relationships between anxiety symptoms and specific cortico-subcortical amyloidosis in older adults, patients with Alzheimer's Disease (AD), and APOE ε4 carriers, but evidence regarding panic attacks is scarce (13, 14). Unfortunately, in this patient, amyloid deposition imaging tests were not conducted. Mixed pathology (i.e., with additional Alzheimer-type pathology) is frequently reported in patients with DLB (2) and is linked to cognition, hallucinations, and survival (15), but evidence regarding the role of amyloidosis in prodromal stages of DLB is lacking. From a clinical standpoint, the qualitative aspects of the patient's panic attacks oriented us toward a panic attack linked to psychiatric-onset DLB rather than events related to Alzheimer's Disease. Thus, anxiety/panic symptoms in DLB interfere severely with daily living (as reported by both patients and informants), while Alzheimer's Disease symptoms rarely interfere and are usually triggered by becoming aware of cognitive deficits (3).

### Therapeutic Intervention

Before index admission, the patient was under rivastigmine transdermal patches (9.5 mg/24 h), Levodopa + Benserazide (750 mg/day, in 4 doses), Pramipexole (0.5 mg/day), Flupentixol-Melitracen (10.5 mg/day), Mirtazapine (15 mg/day), and Estazolam (1 mg/day).

Flupentixol-Melitracen, Mirtazapine, and Estazolam were gradually discontinued, and Quetiapine tablets (12.5 mg/day) were added. Levodopa + Benserazide was reduced to 625 mg/day in 4 doses, and Selegiline was added but not tolerated. Rasagiline was also tried but was found ineffective. At that time, the frequency of panic attacks increased (2–4 per day). Then, Lorazepam (1.5 mg/day) was indicated but discontinued due to fatigue, drowsiness, and unsatisfactory control of symptoms frequency. We could not rule out that the augmented frequency of panic attacks was associated with the change in levodopa daily dose. Evidence in this regard is not conclusive and does not reflect causal mechanisms, but observations show increased anxiety/panic symptoms linked to the OFF phase and a reducted dose of antiparkinsonian drugs (16–18). Therefore, the Levodopa + Benserazide was increased to the previous schema with slight improvement. Gabapentin (1,200 mg/day) was added on subsequent days in conjunction with Sodium Valproate injections (200–400 mg/day) to fully improve panic symptoms. Reduction in panic attacks' frequency was observed, and Sodium Valproate injection was gradually discontinued to avoid worsening of motor symptoms. As panic attacks became shorter and less severe, Trazodone (25 mg/day) was prescribed upon discharge.

### Follow-Up and Patient Perspective

According to her husband, the most severe problems were functional/cognitive decline and panic attacks. Hallucinations and depression symptoms were not considered major issues.

Follow-up treatment recommendations included: the gradual increase of Levodopa + Benserazide dose due to pronounced parkinsonism (until effect or side effects), increase of Rivastigmine patches to 13.3 mg/24 h (plus Memantine if currently tolerated) for dementia, withdrawing of Quetiapine as there were no more significant psychotic symptoms, gradually increasing Trazodone for depressive symptoms, and panic attacks (Venlafaxine or Sertraline if panic symptoms become prominent again), sleep hygiene, and withdrawal of benzodiazepines due to excessive sleep during the day. Multimodal non-pharmacological strategies were also recommended, including physical activity/exercise and cognitive and social stimulation. Once lorazepam was discontinued before discharge, the ambulatory Trazodone (50 mg/day) and Sertraline (50 mg/day) treatments were followed by the total remission of panic attacks. Both memantine and a higher dose of antiparkinsonian medication were not tolerated by the patient as referred by the proxy.

## Discussion

This case report shows that onset of panic attacks in late life can be the first symptom of DLB, thus supporting the notion of a “psychiatric-onset DLB” phenotype as proposed by the prodromal DLB Diagnostic Study Group (2). We describe 5 years of progressing symptoms from the prodromal stage of DLB to dementia, along with the therapeutic interventions considered along the course of the disease. As reports of psychiatric-onset prodromal DLB are limited, the clinical presentation of this case has been detailed to help clinicians understand the clinical picture of this phenotype.

Expert consensus has remarked current challenges in the identification of patients with prominent late-onset psychiatric symptoms at risk of DLB progression (2). Up to one-quarter of patients in the early and prodromal stages of DLB may present anxiety or depression (4). However, preliminary evidence regarding a predominant phenotype (anxiety vs. depression) in patients with psychiatric-onset DLB is not conclusive (19, 20). It has been noted that identification of depression symptoms as a prodromal symptom of DLB may be difficult given the high prevalence of depressive symptoms in older adults (4). By contrast, prior studies have highlighted the importance of prodromal anxiety. Thus, a combination of one core clinical criterion of DLB and prodromal anxiety symptoms has provided a high specificity to separate patients with DLB and patients with Alzheimer's Disease before the onset of dementia (3).

We note that panic attacks have not been explicitly considered in the research criteria for the diagnosis of prodromal DLB. However, the DLB expert consensus has recognized anxiety symptoms (a cluster that includes panic attacks) in psychiatric-onset DLB and suggested expanding the available evidence (2).

Overall, panic attacks are more frequent in women, but the prevalence and incidence of panic attacks decrease in late life. Thus, reported prevalence rates are <0.5% in older adults (over 65 years of age) compared to 1.3% (or greater) in those between 15 and 45 years old (i.e., peak prevalence age for the first episode of panic attacks) (11, 21). Therefore, *de-novo* panic attacks in older adults might represent a diagnostic challenge, and differential diagnosis with non-convulsive epileptic phenomena should be accounted (12). Evidence supports a lower risk of epilepsy in patients with DLB and Parkinson's Disease Dementia when compared to other types of dementia (22), but how recent reports have shown a high frequency (up to 50%) of new-onset seizures around the time of DLB diagnosis (three years before and up to 5 years after) (23). In this case report, some aspects were in favor of an epileptic origin, particularly, the augmented frequency of panic attacks after the administration of quetiapine (which has been reported to lower the seizure threshold and increase the frequency of seizures in dementia) (24, 25), and the partial response to valproate and gabapentin. Nevertheless, we considered a non-epileptic origin because of attack duration, triggering situations that would unlikely trigger partial seizures, and response to antidepressants (partial at the onset of attacks, and total during follow-up with no anticonvulsive medication). Also, a normal 24-h EEG (including panic attacks) supports this diagnosis, although we note that the patient was taking gabapentin and valproic acid, which could confound the interpretation by mitigating abnormal EEG patterns.

Considering the limitations of preliminary studies in patients with psychiatric-onset DLB with anxiety as the first symptom (3), we used structured criteria for both DLB and panic attack diagnosis. The diagnosis of probable DLB was supported by both clinical features and biomarkers following operationalized criteria that provide a biologically based diagnosis (1), while the panic attacks diagnosis was based on DSM-5 criteria (11). Also, symptomatic pharmacological interventions were reported as prior publications have not described in detail treatment responses and side effects in psychiatric-onset DLB, particularly those patients with panic attacks. Finally, for the sake of clear and transparent data reporting, we implemented the Case Report (CARE) guidelines standards (26). Despite the above, some flaws of this report need to be remarked, such as recall bias, use of retrospective clinical data in part of the report, and limited generalizability, that is inherent in case report studies. Moreover, the non-pharmacological and pharmacological interventions were focused on the management of core DLB symptoms and relevant patient symptoms. In addition, treatment was individualized for this particular case (details in general optimal treatment considerations can be found elsewhere) (1). Thus, the authors encourage further research on this population.

In conclusion, although there is no current consensus on how to identify patients with late-onset psychiatric symptoms due to prodromal DLB, this case report can testify that onset of panic attacks in old age can represent the early stage of DLB.

Clinicians and researchers in mental health and related settings need to be aware of this and follow-up patients when atypical and severe psychiatric symptoms are present in older adults to improve diagnosis and expand research.

## Data Availability Statement

The original contributions presented in the study are included in the article, further inquiries can be directed to the corresponding authors.

## Ethics Statement

The case report was reviewed and approved by the Ethics Committee of Shenzhen Kangning Hospital. The patients/participants provided their written informed consent to participate in this study. Written informed consent was obtained from the individual(s) for the publication of any potentially identifiable images or data included in this article.

## Author Contributions

AJ-J: conception of work, methodology, preparation of the initial draft, and writing, reviewing, and approval. DW, DA, and LB: supervision, conception of work, and writing, reviewing, editing, and approval. MB: conception of work, preparation of the initial draft, and writing, reviewing, editing, and approval. HR, JW, QZ, ZX, YY, and PR: conception of work, data acquisition, and writing, reviewing, editing, and approval. All authors contributed to the article and approved the submitted version.

## Funding

This work was supported by the Sanming Project of Medicine in Shenzhen (SZSM201812052) and Guangdong Natural Science Foundation for Major Cultivation Project (2018B030336001). This article also represents independent research partly funded by the Norwegian government—Helse Vest, the National Institute for Health Research (NIHR) Biomedical Research Centre at South London, Maudsley NHS Foundation Trust, and King's College London—U.K.

## Author Disclaimer

The views expressed are those of the authors and not necessarily those of the aforementioned institutions.

## Conflict of Interest

The authors declare that the research was conducted in the absence of any commercial or financial relationships that could be construed as a potential conflict of interest.

## Publisher's Note

All claims expressed in this article are solely those of the authors and do not necessarily represent those of their affiliated organizations, or those of the publisher, the editors and the reviewers. Any product that may be evaluated in this article, or claim that may be made by its manufacturer, is not guaranteed or endorsed by the publisher.
